# The role of LncRNA-mediated autophagy in cancer progression

**DOI:** 10.3389/fcell.2024.1348894

**Published:** 2024-06-12

**Authors:** Zi-yuan Liu, Jia-ming Tang, Meng-qi Yang, Zhi-hui Yang, Jia-zeng Xia

**Affiliations:** ^1^ Gastroenterological Surgery, The Affiliated Wuxi No. 2 People’s Hospital of Nanjing Medical University, Wuxi, China; ^2^ Department of General Surgery, Jiangnan University Medical Center, Wuxi, China; ^3^ Department of Neurology, The Affiliated Wuxi No. 2 People’s Hospital of Nanjing Medical University, Wuxi, China

**Keywords:** lncRNA, cancer, autophagy, tumor progression, chemoresistance

## Abstract

Long non-coding RNAs (lncRNAs) are a sort of transcripts that are more than 200 nucleotides in length. In recent years, many studies have revealed the modulatory role of lncRNAs in cancer. Typically, lncRNAs are linked to a variety of essential events, such as apoptosis, cellular proliferation, and the invasion of malignant cells. Simultaneously, autophagy, an essential intracellular degradation mechanism in eukaryotic cells, is activated to respond to multiple stressful circumstances, for example, nutrient scarcity, accumulation of abnormal proteins, and organelle damage. Autophagy plays both suppressive and promoting roles in cancer. Increasingly, studies have unveiled how dysregulated lncRNAs expression can disrupt autophagic balance, thereby contributing to cancer progression. Consequently, exploring the interplay between lncRNAs and autophagy holds promising implications for clinical research. In this manuscript, we methodically compiled the advances in the molecular mechanisms of lncRNAs and autophagy and briefly summarized the implications of the lncRNA-mediated autophagy axis.

## Introduction

As a heterogeneous group of non-protein coding transcripts, lncRNAs are not only poorly conserved but they can also controlling gene expression at different levels, such as the chromatin, transcriptional, and posttranscriptional levels (Liu et al., 2021). Historically, lncRNAs were dismissed as genomic “junk” and not accorded serious consideration (Statello et al., 2021). However, accumulating evidence now underscores that lncRNAs, in modulating transcription and translation, take a significant operational role. They act as sponges for microRNAs (miRNAs), bound to RNA-binding proteins (RBPs), function as scaffold for proteins, regulate transcription, and even serve as translation templates for peptides (Ransohoff et al., 2018; Ouyang et al., 2022). With increasing evidence pointing to their role in many diseases, especially cancer, lncRNAs are attracting more and more attention.

Autophagy is observed in nearly every type of eukaryotic cells and is a pervasive and highly conserved catabolic process (Zhou et al., 2022). This degradative process is central to cellular regulation, maintaining homeostasis by lysosomal breakdown of injured organelles and various proteins (Chen et al., 2021; Wang et al., 2021). To date, three forms of autophagy have been described: macroautophagy, microautophagy and chaperone-mediated autophagy, the latter of which is only found in mammalian cells (Ktistakis and Tooze, 2016; Xu et al., 2020). Under hypoxic, stressed and deprived conditions, phagophore begins to form and gradually matures into an autophagosome. It then fuses with a lysosome, resulting in the degradation of internal contents within the autolysosome and triggering macroautophagy (Jiang et al., 2021). Microautophagy involves the direct engulfment of small pieces of cytoplasm by the invagination of the lysosomal membrane, which is followed by lysis and subsequent degradation. Unlike the other types, chaperone-mediated autophagy does not entail membrane reorganization. It hinges on the recognition of substrate proteins containing KFERQ motifs, which then bind with cytosolic Hsc70 and cochaperones. This combination is translocated directly across the lysosomal membrane after binding to lysosomal Lamp-2A (Feng et al., 2014; Galluzzi and Green, 2019). Macroautophagy holds the utmost significance among autophagic pathways. For the purposes of the review, our focus will be on macroautophagy, hereafter referred to simply as autophagy.

Cancer is a major burden on socio-economic development and one of the world’s public health problems. In the US, there are projected to be 1,958,310 new cases of cancer and over 609,820 cancer-related deaths in 2023 (Siegel et al., 2023). While recent decades have witnessed remarkable progress to diagnose and treat cancer at an early stage, which has led to a significant reduction in both the number of new cases and the number of deaths, the decline in cancer mortality rates has plateaued worldwide since the 1990s. This stagnation is primarily attributed to the absence of groundbreaking therapies that promise improved prognoses for cancer patients in recent years (Sung et al., 2021). Consequently, there is an urgent requirement to discover novel mechanisms illustrating the etiology of cancer. Both lncRNAs and autophagy play integral roles in a wide array of biological activities and intricate signaling pathways. The existence of potential links between these two key regulatory mechanisms has already been established by mounting explorations (Bermúdez et al., 2019; Islam Khan et al., 2019). Therefore, summarizing the current knowledge on lncRNAs and autophagy is the aim of this review. We will discuss how lncRNAs induce and modulate autophagy in cancer and discuss the implications for clinical applications.

## Overview of LncRNA

**FIGURE 1:**
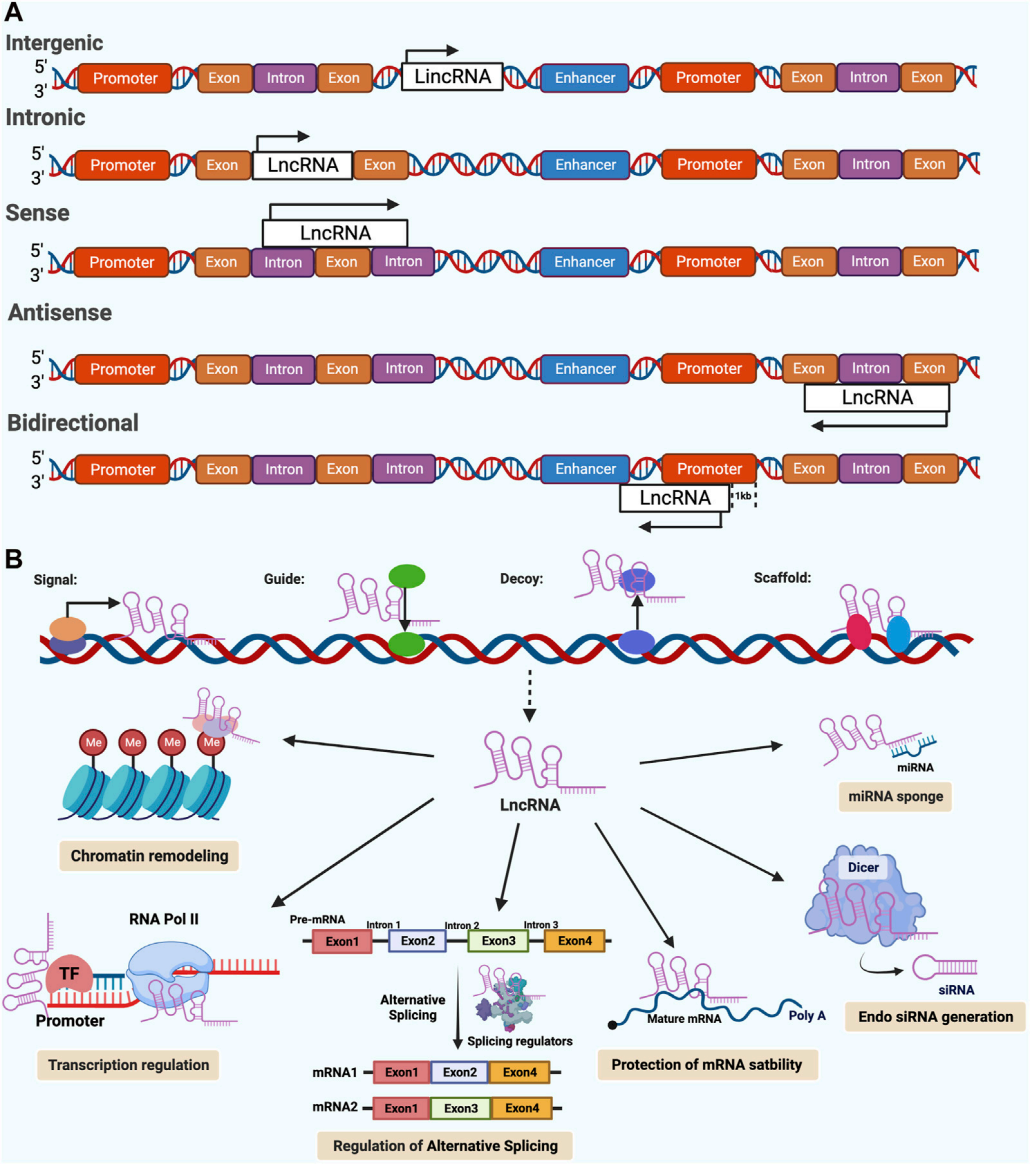
Classifications and functions of Long noncoding RNAs (lncRNAs) in cancer. [modified image from Abdelrahman M. Elsayed et al. (Elsayed et al., 2020)] **(A)** A schematic diagram showing the classification of lncRNAs according to their orientation and position, including intergenic, Intronic, sense, antisense, bidirectional lncRNAs. The arrow indicates the direction of transcription. **(B)** The biological functions of lncRNAs are generally categorized into four main archetypes of molecular mechanisms, including signals, guides, decoys, and scaffolds. Furthermore, lncRNAs have a variety of regulatory functions that are derived from these archetypes, such as; chromatin remodeling, regulation of transcription, alternative splicing and other post-transcriptional modifications, generation of endo siRNA, d protection of mRNA stability, miRNA sponges.

The truth is that less than 2 per cent of the human genome is made up of genes that code for proteins, with about 98 per cent of the rest being transcribed into RNA that does not code for proteins (Djebali et al., 2012; Lin et al., 2020). LncRNAs are mostly transcribed by RNA pol II, and sometimes by RNA pol III, and within the confines of some plant cells, by RNA pol IV and RNA pol V (Smolarz et al., 2021). Many lncRNAs, in addition to those derived from larger precursors, exhibit a conservative and stable protein secondary structure due to a 5′-end cap and 3′-end polyadenylation (e.g., intronic lncRNAs) (Nair et al., 2020; Smolarz et al., 2021). LncRNAs can be classified into five different groups according to the structure of the gene and the position of the gene relative to the protein-coding gene (Figure 1A): (1) sense lncRNAs or (2) antisense lncRNAs, which overlap with neighboring transcripts either in the same direction or in the opposite direction; (3) bidirectional lncRNAs, having their transcript start positions in common with the gene that codes for the protein on the opposite strand; (4) intronic lncRNAs, where the entire lncRNA transcript resides within a coding gene’s intron, and (5) intergenic lncRNAs, which are located in the interval of the genome between two genes (Lin, 2020; McCabe and Rasmussen, 2021).

Since lncRNAs can be detected in the nucleus, cytoplasm or both, their functions are strongly dependent on their subcellular localization (Ransohoff et al., 2018). LncRNAs in the nucleus have a wide range of functions, including interacting with chromatin, regulating transcription and processing RNA. Conversely, lncRNAs in the cytoplasm may control the stability and translation of mRNAs and Impacts on cell signaling pathways (Chen et al., 2018). While the biological functions of lncRNAs are diverse, they tend to fall into four major categories according to their specific mechanisms of action (Figure 1B): signals, guides, decoys and scaffolds (Wang and Chang, 2011; Barangi et al., 2019). The signal lncRNAs are the ones that can be used as markers of notable biological events because they respond to certain stimulus. LncRNAs with guiding functions regulate target gene expression by binding to specific regulators, such as transcription factors and modifiers of chromatin, precise gene targeting for regulation in specific genomic regions (Kashi et al., 2016; Lin, 2020). In contrast to the guidance function, decoys are a class of lncRNAs that bind to or sequester miRNAs and negatively modulate the levels of specific genes, RNA-binding proteins and transcription factors (Wang and Chang, 2011). The most complex molecular archetype of lncRNAs is the scaffolds. They serve as operating workbenches upon which distinct effector molecules interact with each other, influencing their abilities to interact with various partners, ultimately resulting in either transcriptional repression or activation (Huang and Yu, 2015; Schmitz et al., 2016). Interestingly, lncRNAs are known to function in many different ways, and a single lncRNA may perform more than one archetypal function (Elsayed et al., 2020).

Inflammation is thought to play a major role in causing Cancer. Infection and chronic inflammation are responsible for about 25% of all cancers (Murata, 2018). In the 19th century, the German physician Rudolph Virchow first discovered and described the phenomenon of inflammatory cells infiltrating tumors (Rani et al., 2019). In the course of time, scientists discovered the existence of an inflammation-associated microenvironment consisting of cancer cells, immune cells, and several cytokines. These immune cell and cytokine abnormalities complicate the tumor microenvironment (TME), allowing inflammation to play a role in promoting or inhibiting tumor development (Hussain and Harris, 2007; Elinav et al., 2013). Tumor-induced inflammation is a dynamic process in which immune cells are infiltrated and activated. Accumulating evidence suggests that lncRNAs play an important role in this process. They are potent factors in the recruitment and activation of immune cells to regulate tumor development (Xu and Gewirtz, 2022).

In the past few years, with the continuous development of high-throughput sequencing technology, especially the groundbreaking lncRNA microarray and transcript sequencing (RNA-seq), scientists have been able to make enormous progress in the analysis of biomolecules. The latest statistics show that the number of identified human lncRNAs now exceeds 173,000 (Kopp and Mendell, 2018; Zhao et al., 2021). In the complex regulatory network of cancer, lncRNAs have been shown to play a critical role (Yan and Bu, 2021). Similar to other non-coding RNAs, lncRNAs can regulate gene expression through a variety of mechanisms, acting as either cancer-promoting or cancer-suppressing factors, which in turn can regulate the development and progression of cancer. lncRNAs can act as decoys for miRNAs, functioning as competitive endogenous RNAs (ceRNAs) that bind directly to miRNAs, preventing miRNAs from affecting downstream target mRNAs and thus maintaining their functional integrity (Wang et al., 2019). They are also able to influence the activation or repression status of target genes by fine-tuning the interaction between transcription factors and promoters. Furthermore, lncRNAs can act as scaffolding and RNA-binding proteins (RBPs) to directly participate post-transcriptional regulation, modulate protein-protein interactions and several related downstream signaling pathways (Bhat et al., 2016).

### Dual role of autophagy in cancer

There has been evidence that autophagy plays a dual role in cancer, both promoting and inhibiting cancer growth and progression, since the earliest studies of cancer and autophagy (Amaravadi et al., 2019). Autophagy is one of the most important homeostatic mechanisms within the cell. In addition to responding to and alleviating various forms of cellular stress, such as starvation, organelle damage, and redox disturbances, it also contributes to cellular nutrient utilization and promotes metabolism (Debnath et al., 2023). For example, autophagy inhibits tumor growth through the removal of damaged mitochondria and the reduction of reactive oxygen species to inhibit glycolysis. Therefore, it is generally accepted that autophagy is degraded and recycled to inhibit tumor development (Russell and Guan, 2022). However, as cancer develops, the process of autophagy has been shown to be necessary to support uncontrolled growth and progressively increased metabolic activity of tumor cells, leading to tumor dependence on autophagy (Miller and Thorburn, 2021; Qiu et al., 2023). At this stage, aberrant autophagy may contribute to tumor cell proliferation and ongoing tumor progression by promoting cancer stem cell spreading (Ariosa et al., 2021a).

Studies of the BECN1 gene, which encodes beclin-1, provided the first evidence that autophagy plays a tumor suppressor role (Debnath et al., 2023). In the 1990s, Levine’s group identified BECN1 as a tumor suppressor, providing the first insight into autophagy’s role in cancer. Since then, a great deal of research has been undertaken on the role of autophagy in cancer, and it has been found that autophagy plays a cytoprotective role by maintaining cellular and genomic integrity during the early stages of tumor progression, thereby inhibiting tumor development (Ariosa et al., 2021a). Furthermore, ATG proteins have been reported as potential tumor suppressors. In experiments by Marsh et al. knockdown of Atg5 or Atg12 resulted in increased metastasis of cancer cells in a mouse model of breast cancer. This demonstrates a specific role for autophagy in suppressing cancer cell metastasis (Marsh et al., 2020). Eliminating the autophagy protein ATG4C, which is responsible for forming autophagosomes, also plays a role in suppressing tumors (Fu et al., 2019).

### Molecular mechanisms of autophagy

**FIGURE 2:**
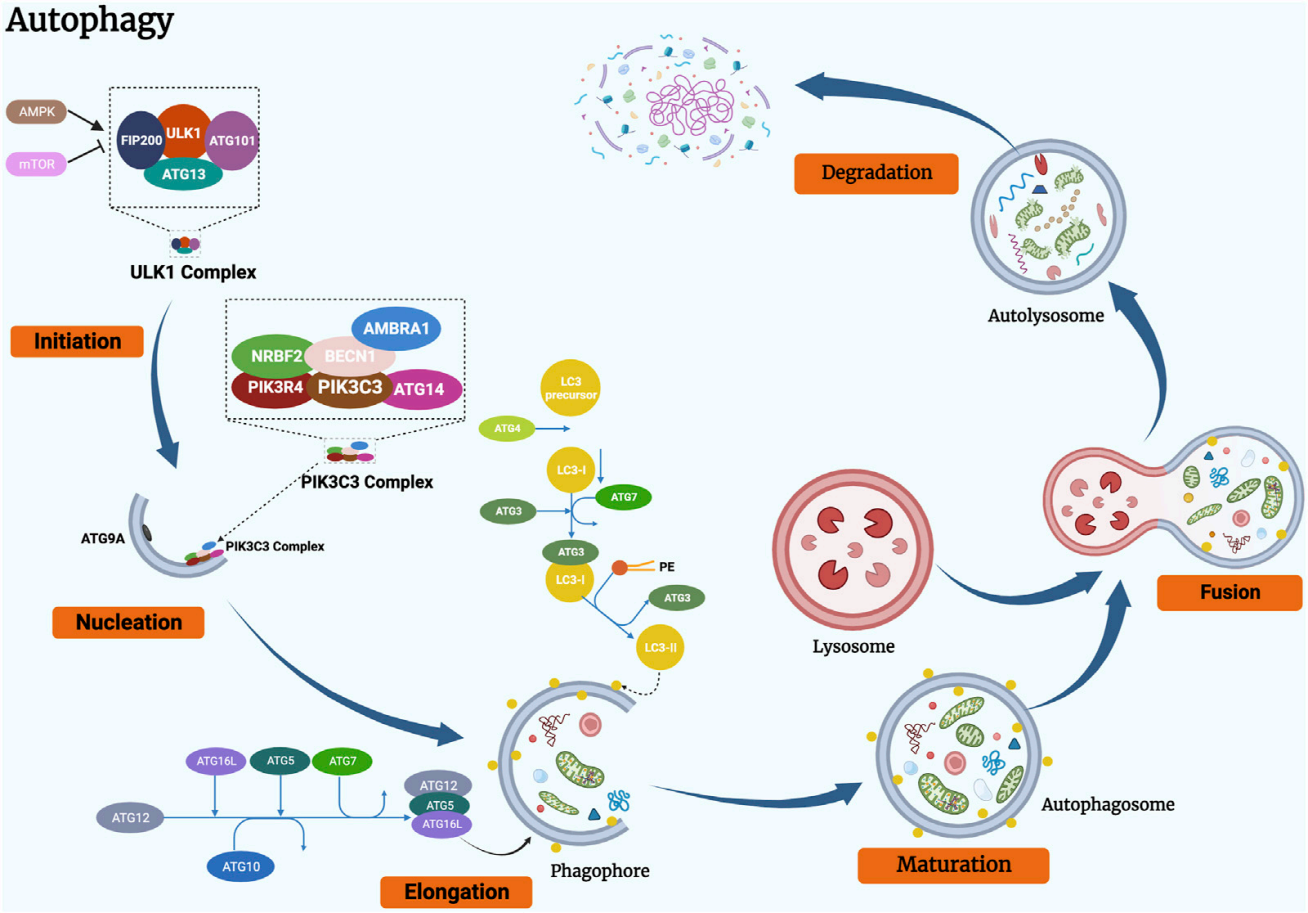
Schematic representation of the basic mechanism of autophagy. The process of autophagy can be divided into the following stages: (1) initiation, (2) vesicle nucleation, (3) vesicle elongation and maturation, (4) vesicle fusion, and (5) cargo degradation.

For autophagy, the process is complex and consists of several consecutive steps: (1) Initiation: the induction of autophagy; (2) Nucleation: the formation of the nucleus of the phagophore; (3) Elongation: during this stage, the phagophore expands, seals, and evolves into the autophagosome; (3) Fusion: autophagosome fusion with a lysosome; (4) Degradation: internal degradation of the material and recycling (Cao et al., 2021; Bai et al., 2022). Figure 2 shows the process and basic mechanism of autophagy.

Autophagy is initiated under a variety of stress conditions (e.g., starvation, hypoxia and oxidative stress) by the release of signals that lead to the activation of the ULK1 complex, which consists of the central kinase protein ULK1, ATG13, FIP200 and ATG101 (Klionsky et al., 2021; C. W; Yun and Lee, 2018). AMPK and mTORC1 regulate autophagy induction (Ktistakis and Tooze, 2016; Han et al., 2022). Under nutrient-rich conditions, mTORC1 is a repressor of autophagy, which can bind ULK1 and avoid autophagy initiation. While AMPK acts as autophagy promoter that persists the balance between the production and consumption of adenosine triphosphate (ATP) (Ashrafizadeh et al., 2021).

Following the induction of autophagy, the ULK1 complex is phosphorylated and translocated into a specific region of the ER, where it facilitates the formation of the PtdIns3K complex, which consists of PIK3C3/VPS34, PIK3R4/VPS15, BECN1 (beclin 1), AMBRA1, ATG14, and NRBF2 (Wang et al., 2018; Kocaturk et al., 2019). Afterwards, the PtdIns3K complex cooperates with ATG9 (a multimembrane-spanning protein) containing vesicles, triggering nucleation of the phagophore and resulting in the phosphoinositide lipids bound to the membrane being phosphorylated, activating the local production of phosphatidylinositol-3-phosphate (PI3P) at the surface of the characteristic ER (called the omegasome) (Chao et al., 2020; Jing et al., 2020). On the omegasome, PI3P recognizes and attracts PI3P-binding proteins which mark the phagophore assembly sites (PAS), such as DFCP1 and WIPIs (here is WIPI2) (Glick et al., 2010; Smith and Macleod, 2019).

After autophagy is initiated, the phagophore continues to rely on two distinct conjugation systems for phagosomal expansion (Chen et al., 2019). The first is the ATG5 - ATG12 - ATG16L1 conjugative system. It consists of ATG12 (acts as a ubiquitin-like factor), ATG5 (acts as an ATG12 background), ATG16L1, ATG7 (acts as an E1 similar enzyme) and ATG10 (acts as an E2 similar enzyme) (Ariosa et al., 2021b). The second is the ATG8 family of proteins (ATG8s), which includes the MAP1LC3/LC3 subfamily and GABARAP subfamily (act as ubiquitin-like proteins), phosphatidylethanolamine (PE, act as substrate), ATG3 (act as a E2 similar carrier protein), ATG7 (act as a E1 similar enzyme), ATG12-ATG5-ATG16L1 complex (act as a E3 ligase), ATG4 (cysteine protease) (Liang et al., 1999; Zhao et al., 2021). At this stage, ATG12 is in the process of activation by ATG7, transferred by ATG10 and ultimately binds to ATG5 (Das et al., 2021). ATG12 and ATG5 subsequently interact with ATG16L1 to form a multimeric protein complex consisting of ATG12, ATG5 and ATG16 (Zhu et al., 2022). Recent studies indicate that ATG16L1 can directly bind WIPI2 on the omegasome, providing a membrane-binding platform for the ATG12-ATG5 conjugate and enhancing ATG3-mediated ATG8 family protein conjugation (J. Liang et al., 2021). LC3 is expressed as a full-length cytoplasmic protein in the majority of cell types. In such a conjugation, precursor LC3 is proteolytically cleaved by ATG4 (cysteine protease) to release a C-terminal glycine residue and to produce a cytoplasmically soluble free form (this form is termed LC3-I) (Xie et al., 2015; Li et al., 2021). Similar to the activation of ATG7 by ATG12, LC3-I was then activated in an ATP-dependent manner (Ohsumi, 2014). Activated LC3-I is delivered to ATG3 prior to covalent conjugation of the C-terminal glycine of LC3-I to PE lipid and formation of LC3-II (a membrane-bound, lipidated form of LC3), in which the ATG12, ATG5 and ATG16 protein complex functions as an E3 ligase to promote the process (Ashrafizadeh et al., 2021; C; Yun and Lee, 2018). The phagophore progressively elongates and becomes curved into a bowl-like structure which engulfs the cytoplasmic components of the cell, eventually closing to form a spherical autophagosome (Cocco et al., 2020). A key signature of autophagy is LC3-II, which marks the formation of autophagosomes (Ajoolabady et al., 2021). Another associated member of the ATG8 protein family, GABARAP, undergoes an analogous process and GABARAP-II localizes to autophagosomes together with LC3-II (Parzych and Klionsky, 2014). Research shows that LC3 proteins participate in vesicle elongation, while GABARAP is engaged in late autophagosome maturation (Weidberg et al., 2010).

ATG8s are not only critical for phagophore membrane expansion and closure, but also have a capacity that can integrate with components of LC3 interaction region (LIR) as well (Koustas et al., 2022). Furthermore, in selected autophagy, LC3 is mainly participate in sequestering specifically labelled cargo into autophagosomes through cargo receptors that contain the LIR (Parzych and Klionsky, 2014). The autophagosome membrane is sealed into a double-layered vesicle once the specifically labelled cargo has been chosen and bound to the membrane (de la Cruz-Ojeda et al., 2022).

The next stage of autophagy is the fusion of autophagosomes with lysosomes to form autolysosomes, after the phagophore membrane has been sealed and the autophagosome has matured (Glick et al., 2010; Parzych and Klionsky, 2014). Accumulating evidence suggests that the microtubule system and associated motor proteins contribute to the movement and traffic of autophagosomes to lysosomes. Autophagosomes and lysosomes are transported to the perinuclear region along the intracellular microtubule system by dynein-dependent mechanisms (C. Yun and Lee, 2018). In the perinuclear domain, SNARE proteins, the membrane-binding proteins and other fusion-associated protein families, such as LAMP-2 and RABs (RAB five and RAB 7), are involved in autolysosomal formation (Zhu et al., 2022). Specifically, the fusion requires the lysosome and autophagosome to be tethered to each other, which is normally controlled by the RAB7, RAB5 and LAMP-2 proteins (Rakesh, 1868; Jogalekar et al., 2021). SNARE proteins include STX17, SNAP29 and VAMP8. The HOPS complex, the best studied tethering protein, is also involved in this process (Rakesh et al., 2022). The HOPS have been identified to play a critical role in the fusion of autophagosomes with lysosomes by capturing Rab7-containing autophagosomes and binding with other adaptor proteins (Faruk et al., 2021; Rakesh et al., 2022).

Eventually, as the autolysosome forms, the lysosomal proteases break down the internal cargo and return nutrients (e.g., amino acids, fatty acids) to the cytosol for further use in different metabolic processes.

### LncRNA-Mediated P62 dependent autophagy

p62 was the first autophagy adaptor protein to be discovered in a mammalian organism. Shin, who discovered p62, named it Sequestosome 1 (SQSTM 1) because of its ability to form aggregates (Shin, 1998; Liu et al., 2016). However, it was not until Komatsu et al. reported a link between p62 and LC3, and found that p62 mediates the formation of protein aggregates for autophagic turnover, that the functional importance of p62 was appreciated. p62 is made up of 440 amino acids and has a multitude of structural domains with different functions. From the N-terminal to the C-segment, they are Phox-BEM1 structural domain (PB1), ZZ-type zinc finger structural domain, nuclear localization signal 1 (NLS1), tumor necrosis factor receptor-associated factor 6-binding (TRAF6-binding) TB domain, nuclear localization signal 1 (NLS2), export motif (NES), and LC3 interaction region (LIR), Keap1 interaction region (KIR) and ubiquitin-associated structural domain (UBA) (Liu et al., 2016; Cerda-Troncoso et al., 2020).

One of p62’s major roles in autophagy is to bind different types of ubiquitinated cargo, which are delivered to autophagosomes via its UBA domain, then to the lysosome via its LIR domain, and finally to the lysosome via its PB1 domain, leading to degradation (Shin et al., 2020; Kumar et al., 2022). In particular, the PB1 domain is able to polymerise with itself to transform into a dimeric form known as homo-oligomerisation. The dimeric form of p62, however, is essentially inactive with regard to autophagy. Despite this, the PB1 domain can also interact with the autophagy receptor NBR1 or other PB1-containing proteins in a process called hetero-oligomerisation. This promotes the polymerisation of the filamentous form of p62, giving the protein function. This structure is important for targeted delivery of ubiquitinated cargo to the autophagosome (Emanuele et al., 2020). There are also other structural domains in the p62 that play an important role in autophagy. For example, the ZZ-type zinc finger domain binds to hydrolysed protein cargo containing amino terminal arginine residues (Nt-Arg), interacting to promote autophagic degradation of the cargo (Cerda-Troncoso et al., 2020; Hennig et al., 2021).

The level of p62 expression in the cell is influenced by several factors. On the one hand it depends on transcriptional regulation, e.g., products of oxidative stress activation (Nrf2), the Ras/MAPK pathway and the JNK/c-Jun pathway all affect p62 transcription. Starvation and proteasome inhibitors also increase p62 transcription. On the other hand, because p62 is a substrate for autophagy activation, the expression level of p62 decreases as the degree of autophagic response increases (Moscat et al., 2016; Qian and Ding, 2023).

The involvement of lncRNAs in the regulation of p62-dependent autophagy has been demonstrated in many studies. New research has shown that the lncRNA CASC9 is upregulated in oral squamous cell carcinoma (OSCC), and knocking down CASC9 in OSCC cells significantly increases autophagy. There was also a significant decrease in the expression of P62 and other valuable biomarkers (Yang et al., 2019). In hepatocellular carcinoma (HCC) cells, lncRNA RP11-295G20.2 was significantly overexpressed and inhibited autophagy by targeting PTEN. Further studies demonstrated that the lncRNA RP11-295G20.2 binds directly to PTEN and promotes its interaction with the specific adaptor protein p62, which induces PTEN degradation via the autophagosome-lysosome pathway. This means that the lncRNA RP11-295G20.2, which mediates p62-dependent autophagy, plays a central role in the degradation of PTEN via the autophagosome-lysosome pathway (L. Liang et al., 2021). In a recent study, Hu and his team found that the lncRNA MITA1 induces autophagy in HCC827GR cells, promoting resistance to gefitinib. By overexpressing the lncRNA MITA1 in HCC827GR cells, they found that p62 levels decreased and the viability of HCC827GR cells improved subsequently. When HCC827-GR cells were treated with an autophagy inhibitor, lncRNA MITA1-mediated regulation of p62 expression was markedly abolished and the effects of lncRNA MITA1 on cell viability were ameliorated. Thus, it can be shown that the lncRNA MITA1 is able to promote resistance to gefitinib in HCC827GR cells through the induction of p62-dependent autophagy (Hu et al., 2021).

## LncRNA-mediated autophagy in cancers

**FIGURE 3:**
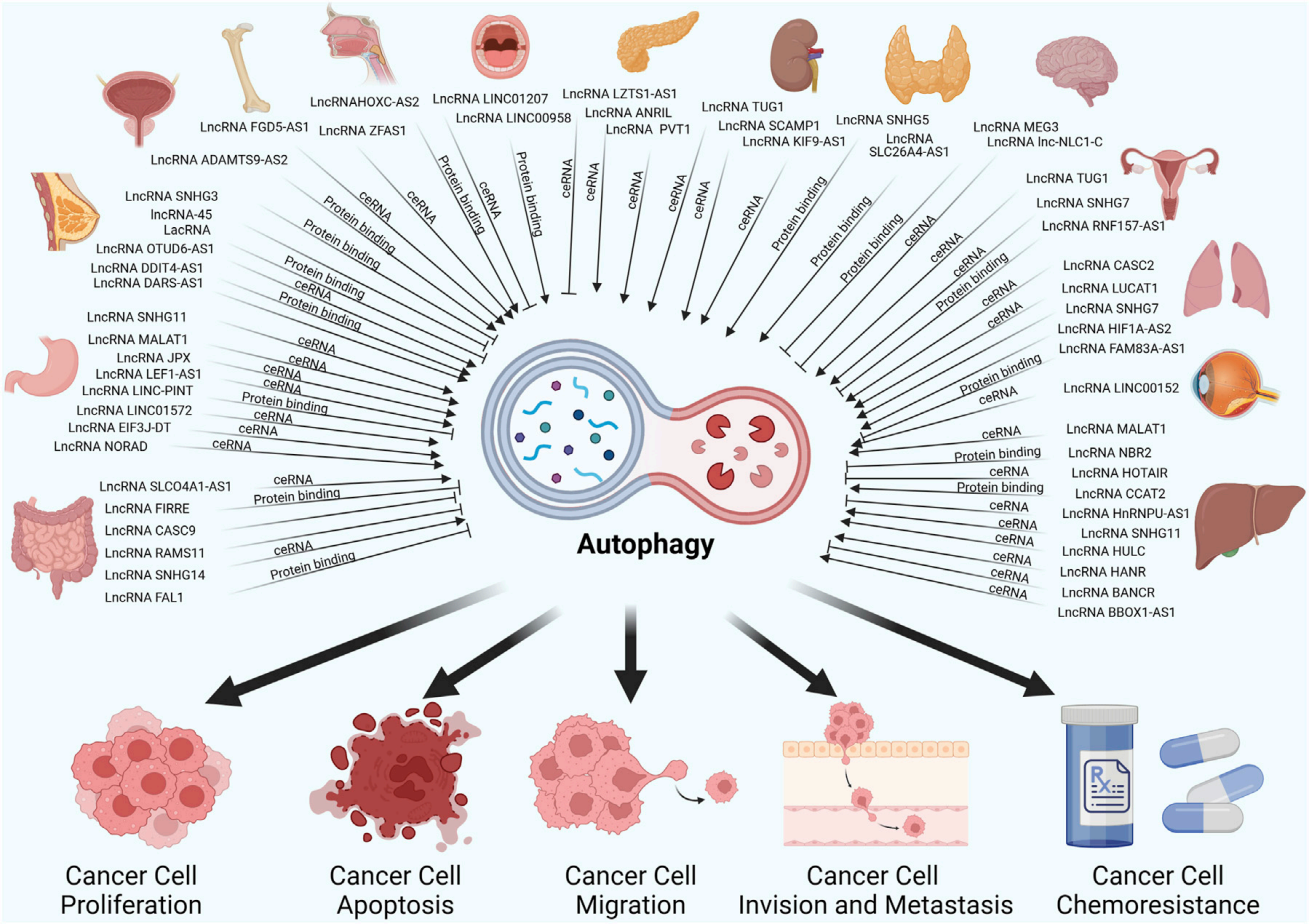
LncRNAs regulate autophagy through different mechanisms thereby affecting cancer cell-associated phenotypes in different cancers.

Many cancers have been reported to have abnormal expression of lncRNAs, and the association between lncRNAs and autophagy has been of particular interest in a variety of cancer types, such as lung cancer, gastric cancer, breast cancer and prostate cancer (Zhang et al., 2021; Zhang L. et al., 2022; Ma et al., 2022). In most of the studies presented, lncRNA regulation of autophagy was mainly mediated by miRNA sponging (ceRNA), RNA to RNA interaction, RNA to protein regulation or some other mechanism (de la Cruz-Ojeda et al., 2022). And these studies show that lncRNAs are involved in several stages of autophagy, from its initiation to its maturation. They mediate the initiation of autophagic phagocytosis by regulating ULK1, mTOR and Beclin-1, and the elongation of autophagic phagocytosis by regulating ATG3, ATG5, ATG4, ATG12 and ATG7. Hence, we have compiled the previous research on the relationships between lncRNA-mediated autophagy and associated phenotypes of cancer cells in Table 1 and Figure 3.

**TABLE 1:** LncRNA-mediated autophagy in cancer progression and chemoresistance.

Function		LncRNA	Role	Expression level	Downstream targets	Autophagy status in cancer	Mechanism	Cancer type
Proliferation and (or) Apoptosis		SNHG11	Oncogene	UP	ATG12	UP	ceRNA	GC
		MALAT1	Oncogene	UP	SIRT1	UP	ceRNA	GC
		MALAT1	Oncogene	UP	LC3	UP	ceRNA	HCC
		NBR2	Suppressor	Down	Beclin 1	Down	Protein binding	HCC
		SLCO4A1-AS1	Oncogene	UP	PARD3	UP	ceRNA	CRC
		FIRRE	Oncogene	UP	PTBP1/BECN1	Down	Protein binding	CRC
		LINC01207	Oncogene	UP	LDHA	Down	ceRNA	OSCC
		LINC00958	Oncogene	UP	Beclin-1/Atg5	UP	Protein binding	OSCC
		HOTAIR	Oncogene	UP	HMGB1	Down	ceRNA	CCA
		LZTS1-AS1	Oncogene	UP	TWIST1	Down	ceRNA	PANC
		TUG1	Oncogene	UP	FLOT1	UP	ceRNA	RCC
		SCAMP1	Oncogene	UP	ZEB1/JUN	UP	ceRNA	RCC
		ADAMTS9-AS2	Suppressor	Down	ADAMTS9	UP	Protein binding	BLCA
		CASC2	Oncogene	UP	ATG5	UP	ceRNA	NSCLC
		SLC26A4-AS1	Oncogene	UP	ETS1/ITPR1	UP	Protein binding	PTC
		SNHG5	Oncogene	UP	FOXO3	UP	Protein binding	PTC
Migration and (or) Invision and (or) Metastasis		SNHG3	Oncogene	UP	AMPK/AKT/mTOR	Down	Protein binding	Breast cancer
		lncRNA-45	Oncogene	UP	mTOR	Down	Not mention	Breast cancer
		LacRNA	Suppressor	Down	PHB2	Down	Protein binding	Breast cancer
		HOXC-AS2	Oncogene	UP	P62	Down	Protein binding	Hypopharyngeal carcinoma
		FAM83A-AS1	Oncogene	UP	AMPKα/ULK1	Down	Protein binding	Lung cancer
		MEG3	Suppressor	Down	FOXO1	Down	Protein binding	Neuroblastoma
		LINC00152	Oncogene	UP	YAP1	UP	ceRNA	Retinoblastoma
		lnc-NLC1-C	Oncogene	UP	PRDX-3	Down	ceRNA	Glioma
		CCAT2	Oncogene	UP	ELAVL1	UP	Protein binding	HCC
		HnRNPU-AS1	Suppressor	Down	SOXS6	Down	ceRNA	HCC
		SNHG11	Oncogene	UP	AGO2	UP	ceRNA	HCC
		JPX	Oncogene	UP	CXCR6	UP	ceRNA	GC
		LEF1-AS1	Oncogene	UP	DEK	UP	ceRNA	GC
		CASC9	Oncogene	UP	AKT/mTOR	Down	Not mention	CRC
		RAMS11	Oncogene	UP	AKT/mTOR	Down	Not mention	CRC
		ZFAS1	Oncogene	UP	ATG10	UP	ceRNA	NPC
Chemoresistance	DDP	LINC-PINT	Suppressor	Down	ATG5	Down	Protein binding	GC
		LINC01572	Oncogene	UP	ATG14	UP	ceRNA	GC
		LUCAT1	Oncogene	UP	ULK1	UP	ceRNA	NSCLC
		SNHG7	Oncogene	UP	LC3B/BECN1	UP	Not mention	NSCLC
		SNHG14	Oncogene	UP	ATG14	UP	ceRNA	CRC
		RNF157-AS1	Oncogene	UP	EZH2/ULK1	Down	Protein binding	Ovarian cancer
	Oxaliplatin	EIF3J-DT	Oncogene	UP	ATG14	UP	ceRNA	GC
		NORAD	Oncogene	UP	ATG5/ATG12	UP	ceRNA	GC
		FAL1	Oncogene	UP	Beclin1	Down	Protein binding	CRC
		HULC	Oncogene	UP	VAMP2	UP	ceRNA	HCC
	Paclitaxel	TUG1	Oncogene	UP	Ago2	UP	ceRNA	Ovarian cancer
		SNHG7	Oncogene	UP	Metformin	UP	ceRNA	Ovarian cancer
		OTUD6-AS1	Oncogene	UP	MTDH	UP	ceRNA	Breast cancer
		DDIT4-AS1	Oncogene	UP	AUF1	UP	Protein binding	Breast cancer
	Sorafenib	HANR	Oncogene	UP	ATG9A	UP	ceRNA	HCC
		BANCR	Oncogene	UP	OLR1	Down	ceRNA	HCC
		BBOX1-AS1	Oncogene	UP	PHF8	UP	ceRNA	HCC
		KIF9-AS1	Oncogene	UP	SMAD3/ATG9A	UP	ceRNA	RCC
	Gemcitabine	ANRIL	Oncogene	UP	HMGB1	UP	ceRNA	PANC
		PVT1	Oncogene	UP	Pygo2/ATG14	UP	ceRNA	PANC
		PVT1	Oncogene	UP	HIF-1α/VMP1	UP	ceRNA	PANC
	Doxorubicin	DARS-AS1	Oncogene	UP	TGF-β/Smad3	UP	Not mention	TNBC
		HIF1A-AS2	Oncogene	UP	HIF-1α/Beclin-1	UP	Not mention	Lung cancer
		FGD5-AS1	Oncogene	UP	WNT5A	UP	ceRNA	Osteosarcoma

### LncRNA-mediated autophagy in proliferation and apoptosis

Firstly, proliferation is one of the most important malignant phenotypes of cancers, and apoptosis holds the same status in neoplastic development. While their functions are opposite and their interactions synergistically control the development of cancers (Evan and Vousden, 2001).

In digestive system cancers, the lncRNA SNHG11 is upregulated in gastric cancer (GC) and is associated with a poor prognosis of the patients, which post-transcriptionally upregulates ATG12 through miR-1276 to enhance autophagy and proliferation and further activate the Wnt/β-catenin signaling pathway (Wu et al., 2021). In a separate study, the lncRNA MALAT1 was shown to increase the autophagic capacity of GC cells by directly targeting miRNA-204 to increase the expression of LC3-II and mediate GC autophagy through the MALAT1/MiR-183/SIRT1 axis (Xu et al., 2021). LncRNA MALAT1 also exhibits different functions in hepatocellular carcinoma (HCC). The expression of the lncRNA MALAT1 was elevated in HCC tissues than in normal tissues. Silencing of MALAT1 promoted HCC autophagy by increasing LC3-II transformation and processing and suppressed HCC cell multiplication (Peng et al., 2020). In contrast, the lncRNA NBR2 acts as a tumor inhibitor in HCC, suppressing Beclin1 and autophagy via ERK/JNK pathways to limit HCC cell proliferation (Sheng et al., 2021). In colorectal cancer (CRC), lncRNA SLCO4A1-AS1 is an oncogenic factor that has a positive correlation with PARD3 and sponges miR-508-3p. SLCO4A1-AS1 promotes CRC cell proliferation and triggers autophagy through the miR-508-3p/PARD3 axis. However, this regulation was disrupted by treatment with 3-methyladenine (3-MA) (Wang and Jin, 2019). Another lncRNA among CRC called firre intergenic repeating RNA element (FIRRE), interacts directly with PTBP1 and enhances BECN1 mRNA stability, thus inducing the reduction of autophagy and promoting CRC cell proliferation (Wang et al., 2022). Significantly, among oral squamous cell carcinoma (OSCC) and cholangiocarcinoma (CCA), upregulated lncRNAs were found to suppress autophagy. In OSCC, high expression of LINC01207 was found in OSCC, and overexpression of LINC01207 promoted the proliferation of OSCC cells, but inhibited apoptosis and autophagy via the miR-1301-3p/LDHA axis (Lu et al., 2021). On the contrary, LINC00958 overexpression could reduce apoptosis and promote autophagy by upregulating the autophagy-related proteins Beclin-1 and Atg5, together with LC3-II/LC3-I ratio, via p53 mediated by SIRT1 (Jiang et al., 2021). Intriguingly, LINC00958 has another pathway to encourage OSCC cells in apoptosis, that is via LINC00958/miR-4306/GSDMD axis. In CCA, lncRNA HOTAIR significantly inhibits apoptotic and autophagic processes and promotes proliferation of CCA cells by targeting the miR-204-5p/HMGB1 axis (Lu et al., 2020). Pancreatic cancer (PANC) is one of the worst-prognostic malignancies, and the molecular mechanisms underlying how it progresses have not been fully elucidated. Wu’s team identified LZTS1-AS1, a highly expressed lncRNA, in PANC cells and tissues, and found that LZTS1-AS1 promotes PANC cell proliferation and inhibits apoptosis and autophagy through the miR-532/TWIST1 axis (Wu et al., 2023).

Among urinary system cancers, nearly 80% of all renal cell carcinomas (RCC) are diagnosed on pathology as clear cell renal cell carcinoma (ccRCC), and which account for 25% of cases, are at risk of developing metastases at an early stage. The group of Dong Lv learned that the high levels of the lncRNA TUG1 associated with ccRCC and confirmed that silencing the lncRNA TUG1 dramatically suppressed cell proliferation and promoted apoptosis, autophagy of ccRCC cells. This was thought to be mediated by the miR-31-5p/FLOT1 axis (Lv et al., 2020). LncRNA SCAMP1 is upregulated in RCC cells and tumor, regulating ZEB1/JUN and autophagy to promote oxidative stress-induced RCC in children through miR-429 (Shao et al., 2019). In bladder cancer (BLCA), the lncRNA autophagy network plays a critical role in BLCA progression. LncRNA ADAMTS9-AS2 is identified downregulated in BLCA and related to ADAMTS9, Zhang et al. showed that the lncRNA ADAMTS9-AS2 inhibits proliferation and elevates autophagy and apoptosis through the PI3K/AKT/mTOR pathway (Zhang et al., 2020).

Downregulation of the lncRNA CASC2 exacerbates NSCLC apoptosis and reduces ATG5-mediated autophagy via regulation of the miR-214/TRIM16 axis in A549 or H1299 NSCLC cells (Li et al., 2018). A reverse autophagy statue was observed in thyroid carcinoma (PTC), Overexpression of lncRNA SLC26A4-AS1 suppressed PTC cells proliferation and promoted autophagy through recruiting transcription factor ETS1 and increasing ITPR1 expression (Peng et al., 2021). Additionally, Qin et al. illuminated that lncRNA SNHG5 was stabilized by RBM47 and targeted to FOXO3, thereby inhibiting proliferation and activating autophagy in PTC cells via the RBM47/SNHG5/FOXO3 axis (Qin et al., 2022).

### LncRNA-mediated autophagy in migration, invasion and metastasis

There is no doubt that the migration, invasion and metastasis of tumor cells are the key factors that lead to the deterioration and eventual death of patients suffering from solid tumors. Although researchers have thoroughly investigated various aspects of tumor growth, we still have little understanding of how lncRNAs and autophagy interact to influence tumor cell migration, invasion and metastasis, and further experimental studies are urgently needed to reveal their exact functions (Guo et al., 2023). In breast cancer (BC), Yu et al. explained how knocking down the lncRNA SNHG3 promotes autophagy by increasing autophagic vacuolization, which inhibits BC cells from migrating and invading (Yu et al., 2023). Analogously, lncRNA-45, a newly identified lncRNA that is transcribed by an internal region within the mTOR complex one gene, is the most upregulated lncRNA. It makes a contribution to BC cells invade and metastasize through activation of mTOR and inhibition of autophagy (Qiu et al., 2023). In addition, LacRNA (a novel LINC00478-associated cytoplasmic RNA) obviously blocked BC cell invasion and metastasis *in vitro* and *in vivo* via inhibiting the degradation of autophagy (Guo et al., 2023).

Hypopharyngeal carcinoma is the most aggressive form of squamous cell carcinoma of the head and neck. Xiang and his team found that the expression of lncRNA HOXC-AS2 and P62 protein in hypopharyngeal carcinoma tissues was significantly higher than that in normal hypopharyngeal tissues. Overexpressing lncRNA HOXC-AS2 could activate the NF-κB signaling pathway through binding to p62 protein, which suppressed the expression of Hmox1 protein, thereby inhibiting the autophagy of hypopharyngeal cancer cells and promoting their migration and invasion (Xiang et al., 2023). Among neurologic tumors, in neuroblastoma (NB), lncRNA MEG3 functions as an anti-tumor agent, and overexpressing MEG3 in NB cells decreased epithelial-mesenchymal transition invasion and metastasis via mTOR signaling and inhibited FOXO1-mediated autophagy (Ye et al., 2020). In retinoblastoma, the investigators observed high levels of LINC00152 expression, and silencing LINC00152 significantly reduced proliferation, invasion and autophagy, while reinforced apoptosis of retinoblastoma cells. Mechanistically, there was evidence that LINC00152 binds directly to miR-613 through ceRNA mechanism and targets YAP1 (Wang et al., 2020). In glioma, lnc-NLC1-C (narcolepsy candidate region one gene C) promotes glioma cell proliferation, migration and invasion and inhibits autophagy via lnc-NLC1-C/miR-383/PRDX-3 axis (Xu et al., 2021).

In lung cancer, the latest scientific findings indicate that the upregulation of lncRNA FAM83A-AS1 expression is not only closely related to the malignancy degree of the tumor, but also directly linked to the poorer quality of survival of patients. Specifically, the knockdown of FAM83A-AS1 gene showed a remarkable effect: it can effectively reduce the proliferation, migration and invasion ability of lung cancer cells. On a deeper level of analysis, the increase in phosphorylated AMPKα and ULK1 that was induced by the knockdown of FAM83A-AS1 revealed a possible molecular signaling pathway. This pathway is likely to involve the MET-AMPKα signaling pathway, which is thought to be an important switch in the control of cellular autophagy. Thus, to some extent, FAM83A-AS1 may contribute to cancer development by inhibiting autophagy through activation of the MET-AMPKα pathway by down-regulating the phosphorylation levels of AMPKα and ULK1. (Zhao et al., 2022). In HCC, Shi et al. revealed that lncRNA CCAT2 functions as an oncogene was upregulated in HCC tissues and cells. Further experiments showed that CCAT-2 was able to modulate miR-4496 and ELAVL-1, thereby inducing the autophagic process, which had the effect of increasing migration and invasion *in vitro* and *in vivo* (Shi et al., 2021). The lncRNA SNHG11/miR-184/AGO2 regulation axis is essential for the promotion of HCC cell proliferating, migrating, invading and autophagy (Huang et al., 2020). In contrast, the group of Li demonstrated that autophagy was shown to prevent invasion and migration of HCC cells. They found that low levels of lncRNA HnRNPU-AS1 positively correlated with bad prediction for HCC patients. Overexpressing HnRNPU-AS1 may constrain the proliferation, migration, and invasion while promoting autophagy in HCC cells via targeting the miR-556-3p and miR-580-3p/SOXS6 axis (Zhang K. et al., 2022). In GC, high lncRNA JPX expression in patients indicates bad prognosis, moreover, knocking down JPX inhibits GC cell activity, invasion and migration by sponging off miR-197, which modulates the downstream CXCR6 protein and promotes autophagy (Han and Liu, 2021). In addition, the lncRNA LEF1-AS1/miR-5100/DEK axis controls GC cell proliferation, invasion and metastasis by promoting autophagy and inhibiting apoptosis by way of the AMPK-mTOR pathway, according to the Zhang et al. study (Zhang et al., 2022).

Epithelial-mesenchymal transition (EMT) pathway takes an elemental role in cancer invasion and migration (Wang et al., 2021; Zhang et al., 2021). LncRNA CASC9 is significantly overexpressed in both the cell lines and tissues of the CRC. CASC9 silencing attenuates cell migration and induces autophagy through AKT/mTOR and EMT signaling pathways. In addition, onco-lncRNA RAMS11 promotes EMT and malignant phenotype of CRC cells by suppression of autophagy and apoptosis in an mTOR-dependent manner (Islam Khan and Law, 2021). A newly identified pseudogene, lnc-CTSLP8, in ovarian cancer which is obviously upregulated in metastatic tumor tissue in comparison to primary ovarian tumors. Mechanistically, lnc-CTSLP8 upregulates CTSL1 and sponges for miR-199a-5p, thereby increasing autophagy and EMT (Xu et al., 2021). In nasopharyngeal carcinoma (NPC), the lncRNA ZFAS1, whose RNA stability is enhanced by the m6A methyltransferase METTL3, promotes NPC cell proliferation, migration and tumor growth and regulates autophagy levels by modulating the miR-100-3p/ATG10 axis and through the PI3K/AKT pathway (Xu et al., 2023).

### LncRNA-mediated autophagy in cancer chemoresistance

There have been multitudinous studies demonstrating the contribution of lncRNAs in the resistance of cancer to drugs and autophagy is increasingly recognized as a critical factor in tumor chemoresistance. LncRNAs can influence treatment-resistant phenotypes through regulation of autophagy, according to recent findings (Xu et al., 2020; Zhang and Lu, 2020; Entezari et al., 2022).

Since 1978, cisplatin (DDP) has been widely used as a first-choice chemotherapy drug to treat approximately half of all solid tumors, such as gastric, breast and lung cancer. The mechanisms that mediate the anti-tumor effects of DDP have been studied for decades, and the most important anti-tumor mechanism is DNA damage through the interaction with the purine bases of DNA (Ranasinghe et al., 2022; Xu and Gewirtz, 2022). However, DDP resistance limits the survival of patients, and a variety of researches reveal that lncRNA-mediated autophagy is one cause of DDP resistance (Xu and Gewirtz, 2022). The LINCRNA PINT/EZH2/ATG5 regulation axis in GC suppresses resistance to DDP by inhibiting the activation of autophagy (Zhang et al., 2022b). Additionally, silencing of LINC01572 inhibits autophagy and resistance to DDP through the miR-497-5p/ATG14 axis in GC cells (Song et al., 2020). In CRC, Han et al. performed that the lncRNA SNHG14/miR-186/ATG14 axis have an important impact on increasing autophagy and facilitating DDP resistance (Han et al., 2020). In NSCLC, the lncRNA LUCAT1 improves resistance to DDP chemotherapy and stimulates autophagy and metastasis of NSCLC cells via targeting the miR-514a-3p/ULK1 axis (Shen et al., 2020). In addition, the upregulated lncRNA SNHG7 promotes NSCLC progression and resistance to DDP by the induction of autophagy activity through the modulation of LC3B and BECN1 (She et al., 2023). In ovarian cancer, elimination of autophagy mediated by DIRAS3 and ULK1 via the lncRNA RNF157-AS1 reduced the resistance of ovarian cancer cell resistance to DDP (Yang et al., 2022). Oxaliplatin is also one of the platinum-based drugs that is commonly treated for cancer, and its resistance is also a major concern (Zhang et al., 2022a). LncRNA EIF3J-DT activates autophagy and contributes to the chemoresistance of oxaliplatin- and 5-Fu-treated cells via miR-188-3p/ATG14 axis among GC (Luo et al., 2021). Coincidentally, lnc-NORAD, which is triggered through both H3K27ac and CREBBP, increased the flux of autophagy in GC cells to repress oxidative stress-induced oxaliplatin resistance by the miR-433-3p/ATG5-ATG12 complex axis (W. J et al., 2021). The lncRNA FAL1, mainly derived from exosomal secretion by CAF, significantly inhibits autophagy induced by oxaliplatin and promotes oxaliplatin chemoresistance in CRC by acting as a scaffold for Beclin1 and TRIM3 to promote the polyubiquitylation of Beclin1 and its degradation (Zhu et al., 2023). In HCC, lncRNA HULC/miR-383-5p/VAMP2 axis promoted the protective autophagy and malignant progression of HCC cells and inhibited the chemosensitivity of oxaliplatin (Li et al., 2021).

5-Fluorouracil (5-FU) is one of the most commonly used chemotherapeutic drugs in the treatment of a wide range of malignancies, including gastrointestinal tumors. 5-FU is an anti-metabolic drug where the hydrogen at the C5 position is replaced by fluorine (Vodenkova et al., 2020). 5-FU interferes with DNA replication by inhibiting the intracellular activity of thymine nucleotide synthetase (TS), and also has some inhibitory effects on RNA synthesis (Ghafouri-Fard et al., 2021; Sethy and Kundu, 2021). LINC01871 was found to be expressed at low levels in CRC tissues and cell lines, and patients with low LINC01871 levels had significantly worse survival. LINC01871 can sponge miR-142-3p and regulate ZYG11B expression to induce autophagy, which increases CRC cell sensitivity to 5-FU and promotes CRC cell chemotherapy resistance (Duan et al., 2023). Similarly, knockdown of lncRNA NEAT1 significantly inhibited CRC cell proliferation, autophagy and enhanced 5-FU sensitivity via targeting miR-34a (Liu et al., 2020). In GC, lncRNA FEZF1-AS1 is upregulated in the tissues of chemotherapy-resistant gastric cancer. Downregulation of FEZF1-AS1 can directly modulate autophagy via ATG5, thereby enhancing multi-drug resistant and improving 5-FU sensitivity in GC cells (Gui et al., 2021).

Paclitaxel (PTX) is also a first-line chemotherapeutic agent for cancer that bonds to and stabilizes microtubules, leading to a disruption of the metaphase-to-anaphase junction during the mitosis process (Zhu and Chen, 2019). Recent evidence reveals a variety of mechanisms for PTX resistance, one of which is autophagic responses mediated by lncRNAs (Gu et al., 2020; Yu et al., 2022). In ovarian cancer, the lnc-TUG1/miRNA-29b-3p/Ago2 axis triggers autophagy and, as a result, leads to PTX resistance (Gu et al., 2020). In a similar investigation, researchers found that metformin could induce the sensitivity of paclitaxel in ovarian cancer by the regulation of lncRNA SNHG7/miRNA-3127-5p-mediated autophagy (Yu et al., 2022). In BC, oncogenic lnc-OTUD6B-AS1 drives resistance to paclitaxel and induces autophagy as well as DNA damage by regulating the miRNA-26a-5p/MTDH signaling pathway (Li et al., 2021). Intriguingly, the lnc-DDIT4-AS1 enhances autophagy and makes BC cells more sensitive to PTX by promoting the interacting DDIT4 mRNA with the AUF1 protein, resulting in inhibition of the mTOR pathway (Jiang et al., 2023).

Sorafenib, a novel multi-kinase inhibitor, is able to selectively disable the relevant kinases and abnormal signaling pathways. It is widely used in the treatment of RCC and HCC. In sorafenib-resistant HCC cells, Shi’s group discovered that the lnc-HANR was significantly hyper-expressed, which increases resistance to sorafenib via promoting autophagy through the axis of miRNA-29b/ATG9A (Shi et al., 2020). I In addition, the lnc-BANCR/miR-590-5P/OLR1 axis inhibited autophagy and promoted sorafenib responsiveness in HCC under the regulation of rutin, the major constituent of Potentilla discolor bunge (Zhou et al., 2021). A comparable study has been executed in HCC cells and proved that the lnc-BBOX1-AS1 enhances PHF8-driven autophagy and the resistance of HCC cells to the drug sorafenib by modulating the miRNA-361-3p/PHF8 (Tao et al., 2022). In RCC, lnc-KIF9-AS1 recruits and fixes miRNA -497-5p, regulates SMAD3, TGF-β and ATG9A-mediated autophagy and thus promotes the resistance to sorafenib (Jin et al., 2020).

Gemcitabine is a cytosine nucleotide analogue that is widely used as an anti-cancer drug to treat a number of conditions, especially PANC (Binenbaum et al., 2015). Wang’s research revealed that lnc-ANRIL is elevated in PANC tissue and that knockdown of ANRIL decreases chemoresistance to gemcitabine through the targeting of miRNA-181a/HMGB1-driven autophagy (Wang et al., 2021). Another oncogenic lnc-PVT1 increases chemoresistance to gemcitabine by sponging off miRNA-619-5p and through activation of the Pygo2/Wnt/β-catenin pathway and ATG14 mediated-autophagy pathway (Zhou et al., 2020). Interestingly, through the axis of miRNA-143/HIF-1α/VMP1, PVT1 knockdown reduces autophagic activity and increases gemcitabine sensitivity in PANC (Liu et al., 2021).

Doxorubicin (DOX) is an antibiotic chemotherapeutic agent which inhibits proliferation and induces apoptosis by blocking topoisomerase II activity and causing DNA breaks (Ashrafizaveh et al., 2021). lncRNA-mediated autophagy is closely in connection with DOX resistance in cancer cells, according to a growing number of studies (Guçlu et al., 2021; Fei et al., 2022). Among triple-negative breast cancer (TNBC), a new type of nanodrug delivery system based on CL4-modified exosomes was able to deliver the siRNA of lnc-DARS-AS1 to TNBC cells, and silencing of DARS-AS1 by this delivery system increased DOX sensitivity of BC cells by suppressing autophagy induced by the TGF-β/Smad3 axis (Liu et al., 2023). In lung cancer, knocking down the lncRNA HIF1A-AS2 made lung cancer cells more sensitive to DOX and reduced autophagy, but the detailed mechanism remained unclear (Guçlu et al., 2021). In osteosarcoma, the lnc-FGD5-AS1 is upregulated in osteosarcoma cells resistant to doxorubicin, and knockdown of its expression attenuates the chemoresistance to DOX by WNT5A-driven autophagy through miRNA-154-5p sponging (Fei et al., 2022).

Therefore, lncRNA-mediated autophagy can be expected to be a major contributor to overcoming chemoresistance in cancer cells. And, focusing on the lncRNA-autophagy axis has promise for accelerating the clinical translation of novel drugs.

## Conclusion and future perspectives

To date, lncRNAs have emerged as a novel approach for investigating various facets of cancer, including detecting, diagnosing, responding to treatment and prognostic values. And the functional analysis of is especially important for understanding chemoresistance. Simultaneously, autophagy ubiquitously present in almost all eukaryotes, playing essential roles in the material homeostasis in cancer cells (Chang et al., 2021).

In this manuscript, we systematically explored the classifications and functions of lncRNAs, the essential cellular processes of autophagy, and the significance of lncRNA-mediated autophagy in the progression of various malignancies and chemotherapy resistance. We conclude that, lncRNAs regulate autophagy mechanisms primarily through two modes: ceRNAs and RBP interactions, as evidenced in the majority of studies. On one hand, lncRNAs modulate autophagy genes expression by binding to specific miRNAs, including proteins from the mTOR, ULK1 and ATG families. . . . . . . These genes are crucial for the activation of related pathways that influence the autophagy process. On the other hand, lncRNAs can bind directly to key proteins involved in autophagy initiation.

Notably, in a previous study, the findings of Liang et al. were quite remarkable. When they investigated the different mechanisms by which lncRNAs regulate autophagy, they found an interesting phenomenon: when lncRNAs work through non-ceRNA mechanisms, they tend to reduce the level of autophagy, but the opposite is often the case when lncRNAs work through ceRNA mechanisms (Yu et al., 2022). However, on the basis of recent studies, we found that lncRNAs also inhibit the level of autophagy through the ceRNA mechanism, such as HOTAIR in CCA, LZTS1 AS1 in PANC. Therefore, researchers urgently need to conduct further studies to better understand the complex and delicate mechanism by which lncRNAs and autophagy interact. These studies should focus on the identification and validation of specific lncRNAs that activate or inhibit the ceRNA machinery, as well as the exploration of the specific molecular details of how they affect autophagy. In addition, the deeper secrets behind the role of lncRNAs in regulating autophagy will be unraveled by combining multidisciplinary approaches such as molecular biology, biochemistry and genetics.

Furthermore, in our opinion, the lncRNA-mediated autophagy axis holds significant value for clinical applications. Primarily, given its deep involvement in the chemoresistance of cancers, targeting this axis could significantly improve chemotherapy efficacy. Secondly, considering the widespread participation of the lncRNA-mediated autophagy axis in anti-neoplastic activity of newly developing drugs, combining therapies that target this axis with these drugs may amplify therapeutic effects. Thus, extensive investigation is required to unravel the complicated interplay between lncRNAs and the complex regulatory autophagy process in order to identify both novel diagnostic biomarkers and potential therapeutic interventions.
